# Farnesoid X receptor, a novel proto-oncogene in non-small cell lung cancer, promotes tumor growth via directly transactivating *CCND1*

**DOI:** 10.1038/s41598-017-00698-4

**Published:** 2017-04-04

**Authors:** Wenjie You, Bi Chen, Xueqing Liu, Shan Xue, Hui Qin, Handong Jiang

**Affiliations:** 1 Department of Respiratory Medicine, Ren Ji Hospital, School of Medicine, Shanghai Jiao Tong University, Shanghai, China; 2 Department of Respiratory Medicine, Affiliated Hospital of Xuzhou Medical University, Xuzhou, China

## Abstract

Farnesoid X receptor (FXR), a nuclear receptor for maintaining bile acid homeostasis, has been recognized as a tumor suppressor in enterohepatic tissues. However, its expression and functional role in non-small cell lung cancer (NSCLC) remain unclear. We report that FXR is significantly increased in NSCLC and that it predicts poor clinical outcomes in NSCLC patients. FXR knockdown in NSCLC cells inhibited *in vitro* cell proliferation, blocked xenograft growth in nude mice, and delayed the G1/S transition of the cell cycle, whereas ectopic overexpression of FXR promoted NSCLC cell proliferation. Mechanistic analysis demonstrated that FXR could directly bind to an inverted repeat-0 sequence in the *CCND1* promoter and activate its transcription. Cyclin D1 overexpression rescued NSCLC cells from the delayed G1/S transition and the impaired cell proliferation induced by FXR knockdown. Importantly, a positive correlation between the expression of FXR and cyclin D1 was confirmed in NSCLC samples, and patients with high expression of both FXR and cyclin D1 had the worst prognosis. In summary, our results suggest that FXR has oncogenic potential in NSCLC development, providing mechanistic insights that could be exploited for both prognostic and therapeutic purposes.

## Introduction

Lung cancer is the leading cause of cancer mortality worldwide, and approximately 85% of cases are non-small cell lung cancer (NSCLC)^1^. Despite major advances in therapeutic strategies, the prognosis for NSCLC patients remains poor; the 5-year survival rate is less than 15%^2^. A growing number of molecular alterations and specific gene expression signatures have been implicated in NSCLC carcinogenesis, including *KRAS*, *EGFR* mutations, *EML4-ALK* rearrangements, and VEGF and thymidylate synthase expression levels, among others^3–5^. However, the detailed molecular pathogenesis of NSCLC is far from fully understood.

The farnesoid X receptor (FXR) is a member of the nuclear receptor superfamily that is predominantly expressed in the liver, intestines, kidneys, and adrenal glands^6, 7^. As a bile acid (BA)-activated transcription factor, FXR maintains BA homeostasis by controlling the transcription of numerous genes involved in BA synthesis, conjugation, and transportation^8^. Small heterodimer partner (SHP), a well-characterized FXR target gene, mediates many of FXR’s pleiotropic functions, such as repressing the transcription of the BA synthetase *CYP7A1* ^9^. Emerging evidence supports an important role for FXR in tumorigenesis, as either an oncogene or a tumor suppressor gene. FXR deficiency in mice has been reported to cause activation of the Wnt/β-catenin pathway in the liver and ultimately to generate spontaneous liver tumors^10, 11^. Other studies have revealed increased colon cell proliferation and small intestine adenocarcinoma formation in FXR^−/−^ mice; FXR expression is inversely correlated with human colorectal cancer progression^12, 13^. Several other studies have demonstrated a causative role for FXR in the carcinogenesis of organs other than the liver-intestine system. FXR has been found to be associated with a higher tumor grade, greater tumor size and lymph node metastasis in esophageal adenocarcinomas, and FXR knockdown has been shown to suppress tumor cell growth both *in vitro* and *in vivo* ^14^. In estrogen receptor (ER)-positive breast cancer, increased FXR levels were correlated with a proliferation marker, Ki-67, and lymph metastasis in postmenopausal women^15^. Therefore, the precise contribution of FXR to carcinogenesis in different cancer types is controversial.

We previously reported that FXR was increased in idiopathic pulmonary fibrosis (IPF) lungs^16^. Beyond that, knockdown of FXR restrained BA-induced epithelial-mesenchymal transition and lung fibroblast activation, indicating a detrimental role of FXR in IPF pathogenesis. However, the expression and potential biological function of FXR in NSCLC have never been investigated yet. Here, we firstly showed that FXR was upregulated in NSCLC tissues, and its expression levels were positively correlated with poor clinical outcomes. We identified an oncogenic role of FXR in NSCLC development by uncovering a mechanistic link to cyclin D1. Furthermore, FXR-cyclin D1 signaling was found to predict a poor prognosis for NSCLC patients. This finding may permit the stratification of NSCLC in a novel way and may subsequently provide an intervention option.

## Results

### FXR is upregulated in NSCLC and predicts poor patient outcomes

**Figure 1:**
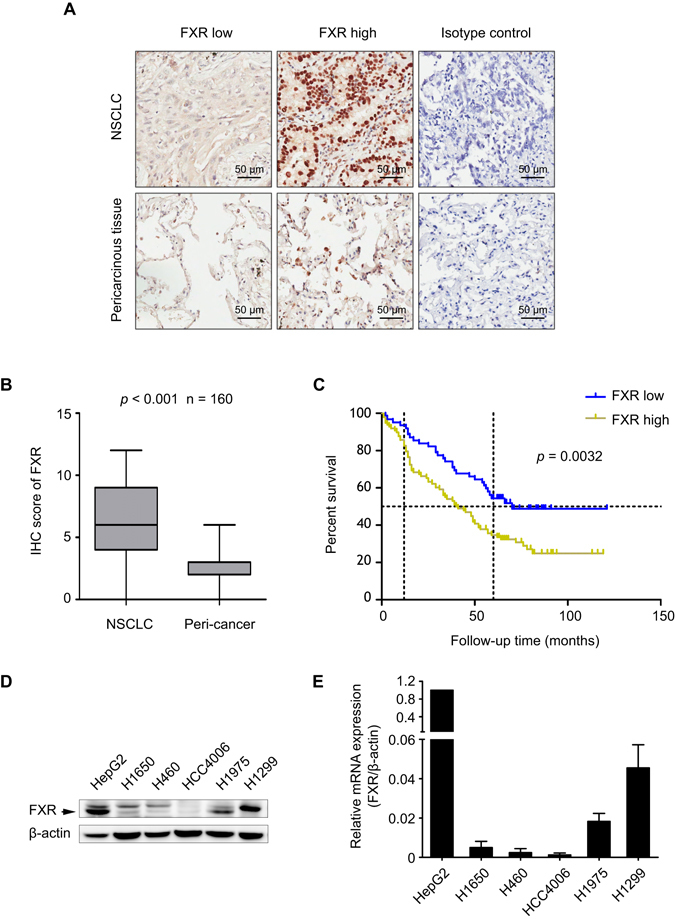
FXR is upregulated in NSCLC and predicts poor outcomes for patients. IHC was performed in 160 pairs of NSCLC and matched normal lung tissues. (**A**) Representative images of FXR high or low expression in NSCLC tissues (upper panel), and images of FXR expression in corresponding pericarcinous lung tissues (lower panel) are shown. Isotype control: the primary antibody was replaced by nonspecific rabbit IgG. (**B**) IHC score of FXR in these paired samples (*p* < 0.001). (**C**) Kaplan-Meier analysis of 160 NSCLC patients showing that “FXR high” patients (n = 98) have a shorter OS than “FXR low” patients (n = 62) (*p* = 0.0032). HepG2 and five NSCLC cell lines were lysed and collected. (**D**) Western blot was performed to evaluate the FXR protein expression. The band for FXR was indicated by an arrow. (**E**) Quantitative RT-PCR analysis of FXR mRNA expression in these cell lines. In HepG2, the expression level of FXR mRNA was set at 1. All experiments were repeated at least three times. β-actin was used as an internal control.

We first assessed the FXR expression level by immunohistochemical (IHC) staining in 160 pairs of NSCLC and matched normal lung tissues. Intense nuclear FXR staining was observed in tumor cells but was rare in normal cells (Fig. 1A). We found that FXR protein was significantly upregulated in carcinous, compared to pericarcinous, lung tissues (Fig. 1B).

For further analysis, the NSCLC specimens were separated into two groups, defined as “FXR high” (scores of 6–12) and “FXR low” (0–4). Consistent with the previous observations, the proportion of “FXR high” specimens in NSCLC was significantly higher than that in matched normal lung tissues (61.2% vs. 0.6%, *p* < 0.001) (Table 1). Statistical data showed that high FXR expression was positively associated with a more advanced pathological stage (*p* = 0.012) and T status (*p* = 0.025), as well as a larger tumor size (*p* = 0.03) (Table 2), indicating the possible involvement of FXR in tumor growth and NSCLC progression.

**Table 1:** Protein expression levels of FXR and cyclin D1 in NSCLC and pericarcinous lung tissues.

Tissue sample	NO. of patients	FXR		*p-*value	Cyclin D1		*p-*value
		Low (%)	High (%)		Low (%)	High (%)	
NSCLC	160	62 (38.8)	98 (61.2)	<0.001^a^	69 (43.1)	91 (56.9)	<0.001^a^
Peri-cancer	160	159 (99.4)	1 (0.6)		152 (95.0)	8 (5.0)	

^a^Data were analyzed using chi-square test.

**Table 2:** Clinicopathologic characteristics according to FXR and cyclin D1 protein expression in NSCLC.

Variables	NO. of patients	FXR		*p-*value	Cyclin D1		*p-*value
		Low (%)	High (%)		Low (%)	High (%)	
Gender							
Male	115	44 (38.3)	71 (61.7)	0.839^a^	52 (45.2)	63 (54.8)	0.393^a^
Female	45	18 (40.0)	27 (60.0)		17 (37.8)	28 (62.2)	
Age (year)							
≤60	61	25 (41.0)	36 (59.0)	0.649^a^	25 (41.0)	36 (59.0)	0.668^a^
>60	99	37 (37.4)	62 (62.6)		44 (44.4)	55 (55.6)	
Stage							
I-IIa	73	36 (49.3)	37 (50.7)	0.012^a^	42 (57.5)	31 (42.5)	0.001^a^
IIb-IV	87	26 (29.9)	61 (70.1)		27 (31.0)	60 (69.0)	
Tumor size (cm)							
≤3.0	51	26 (51.0)	25 (49.0)	0.03^a^	29 (56.9)	22 (43.1)	0.016^a^
>3.0	109	36 (33.0)	73 (67.0)		40 (36.7)	69 (63.3)	
T status							
T1	30	17 (56.7)	13 (43.3)	0.025^a^	18 (60.0)	12 (40.0)	0.038^a^
T2/T3/T4	130	45 (34.6)	85 (65.4)		51 (39.2)	79 (60.8)	
N status							
N0	79	36 (45.6)	43 (54.4)	0.156^a^	36 (45.6)	43 (54.4)	0.537^a^
N1/N2/N3	81	28 (34.6)	53 (65.4)		33 (40.7)	48 (59.3)	
M status							
M0	158	61 (38.6)	97 (61.4)	1^b^	68 (43.0)	90 (57.0)	1^b^
M1	2	1 (50.0)	1 (50.0)		1 (50.0)	1 (50.0)	
NO. of positive lymph nodes							
Mean		1.61	2.22	0.221^c^	1.88	2.05	0.721^c^
SD		2.91	3.64		3.18	3.51	

^a^Data were analyzed using chi-square test. ^b^Data were analyzed using Fisher’s exact test. ^c^Data were analyzed using Mann-Whitney *U* test.

The prognostic implication of FXR in NSCLC was subsequently analyzed. Kaplan-Meier survival curves showed that the overall survival (OS) was significantly worse in “FXR high” NSCLC patients than in those with low expression (*p* = 0.0032) (Fig. 1C). Multivariate Cox regression analysis revealed that an “FXR high” pattern [hazard ratio (HR) 1.71, 95% confidence interval (CI) 1.108–2.637; *p* = 0.015], as well as advanced stage (HR 1.773, 95% CI 1.171–2.685; *p* = 0.007), were independent predictors for poor OS in NSCLC (Table 3).

**Table 3:** Univariate and multivariate Cox regression analysis for OS in NSCLC.

Variable	Univariate analysis			Multivariate analysis		
	HR	95% CI	*p*-value	HR	95% CI	*p*-value
Gender						
Male	1					
Female	1.283	0.845–1.947	0.242			
Age (year)						
≤60	1					
>60	1.11	0.737–1.669	0.618			
Stage						
I-IIa	1			1		
IIb-IV	1.927	1.279–2.905	0.002	1.773	1.171–2.685	0.007
Tumor size (cm)						
≤3.0	1					
>3.0	1.589	1.02–2.476	0.041			
FXR expression						
Low	1			1		
High	1.875	1.222–2.879	0.004	1.71	1.108–2.637	0.015
Cyclin D1 expression						
Low	1					
High	1.678	1.116–2.521	0.013			

### FXR promotes the proliferation of NSCLC cells, and knockdown inhibits tumor growth *in vivo*

The FXR expression level in five NSCLC cells was evaluated by western blot and quantitative real-time PCR (RT-PCR). H1975 and H1299 had higher FXR protein and mRNA expression compared with H1650, H460 or HCC4006 cells (Fig. 1D and E). HCC4006 had the lowest FXR expression.

**Figure 2:**
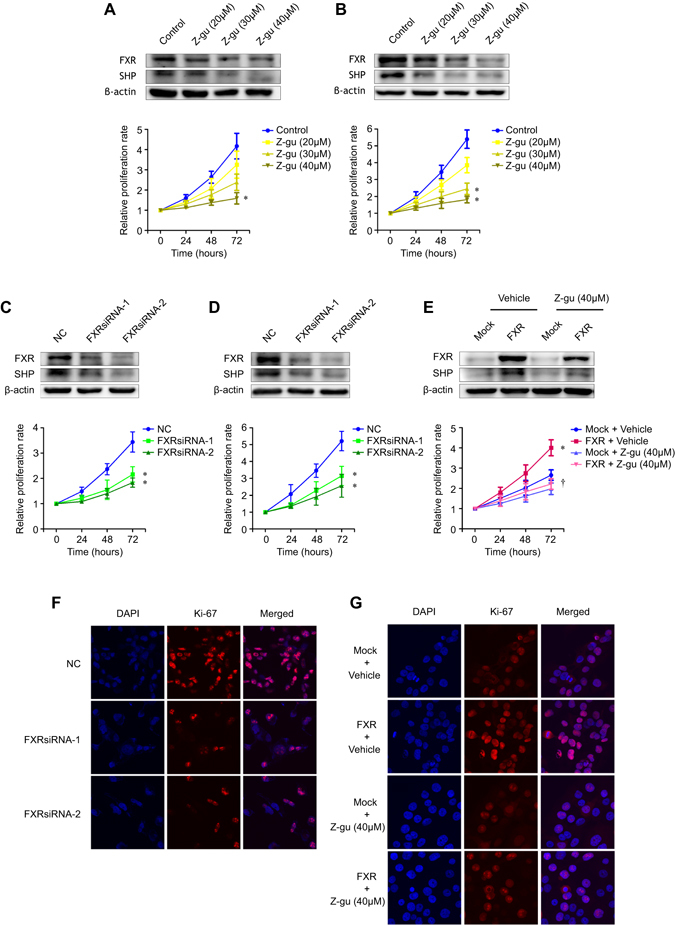
FXR promotes the proliferation of NSCLC cells *in vitro*. H1975 and H1299 cells were treated with Z-guggulsterone (Z-gu, 20, 30, and 40 μM) (**A** and **B**), or were transfected with NC- or FXR-siRNA sequences (**C** and **D**). HCC4006 stable cell lines were treated with Z-guggulsterone (40 μM) (**E**). The FXR and SHP protein expression levels in each cell line were determined by western blot two days later (upper graph). Cell proliferation was determined after the indicated treatment by using SRB assay at different time points (lower curve). Representative images of immunofluorescent staining of Ki-67 in H1975 cells transfected with NC- or FXR-siRNA sequences (**F**), and HCC4006 stable cell lines treated with Z-guggulsterone (40 μM) (**G**) at × 60 magnification. The nuclei were stained with DAPI. All experiments were repeated at least three times. *p < 0.05, compared with the control, NC, or mock group, respectively; ^†^ *p* < 0.05, compared with the FXR-overexpressed HCC4006 cell group.

As the FXR expression level was correlated with poor clinical outcomes, we hypothesized that FXR might act as an oncogene in NSCLC. We used the downstream target, SHP, as an indicator of alterations in FXR activity with treatment^9^. The FXR antagonist Z-guggulsterone inhibited the proliferation of higher-FXR-expressing cell lines, H1975 and H1299, in a dose-dependent manner (Fig. 2A and B). FXR knockdown resulted in decreased proliferation of H1975 and H1299 cells (Fig. 2C and D). By contrast, enforced FXR in endogenous lowest-FXR-expressing cells HCC4006 significantly accelerated cell proliferation compared to a mock group, which was reversed by Z-guggulsterone (Fig. 2E). Ki-67 is a universal biomarker for proliferating cells^17^. Our immunofluorescence data showed that FXR silencing in H1975 compromised the intensity and proportion of Ki-67-positive cells, while ectopic FXR expression in HCC4006 had the opposite effects (Fig. 2F and G).

**Figure 3:**
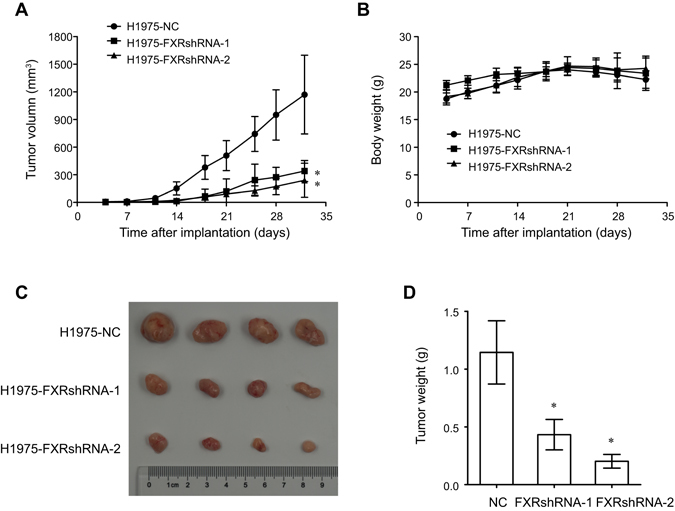
FXR knockdown inhibits NSCLC tumor growth *in vivo*. Stable H1975 cell lines with NC- or FXR-shRNA sequences were subcutaneously injected into the right flanks of nude mice. The tumor volume (**A**) and body weight (**B**) in each group (n = 6) were monitored twice a week after implantation. After 32 days, tumors were isolated to be photographed (**C**) and weighed (**D**). The data are shown as the mean ± SD. *p < 0.05, compared with the NC group.

Next, we evaluated the effect of FXR knockdown on NSCLC xenograft growth *in vivo*. H1975 cells that were transfected with FXR- or NC-shRNA were subcutaneously injected into nude mice. Figure 3A reveals significantly slower tumor growth according to the volumes in FXR knockdown groups compared to the control group. However, no marked difference in body weight was observed (Fig. 3B). After 32 days, tumors were isolated. We found that tumors derived from FXRshRNA-transfected H1975 cells had a significantly smaller tumor size and tumor weight compared to those from control cells (Fig. 3C and D). These data indicated that FXR had proliferation-promoting properties in NSCLC.

### Suppression of FXR causes G0/G1 cell cycle arrest and cyclin D1 reduction, independent of SHP in NSCLC

**Figure 4:**
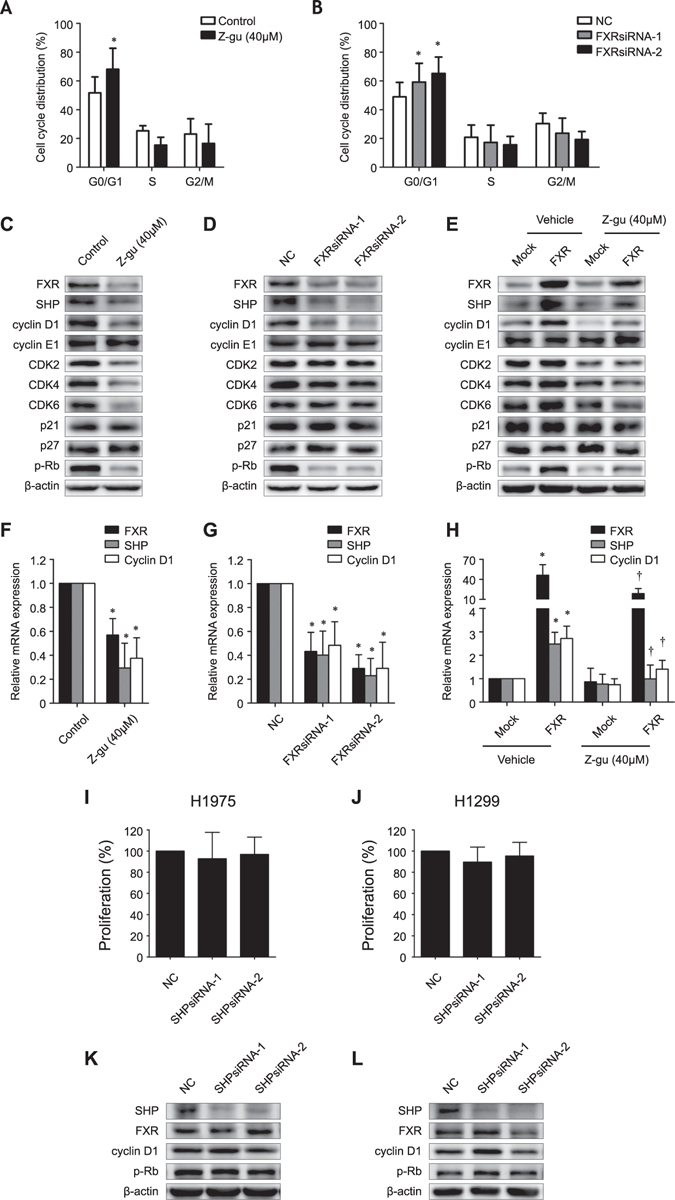
Effects of FXR on cell cycle distribution and cell cycle regulators, independent of SHP in NSCLC. The cell cycle profile of H1975 cells was analyzed by flow cytometry two days after treatment with Z-guggulsterone (Z-gu, 40 μM) (**A**), or transfection with NC- or FXR-siRNA sequences (**B**). Western blot analysis was performed to evaluate the expression of FXR, SHP, cyclin D1, cyclin E1, CDK2, CDK4, CDK6, p21^Cip1^, p27^Kip1^, and p-Rb in H1975 cells that were treated with Z-guggulsterone (40 μM) (**C**) or transfected with NC- or FXR-siRNA sequences (**D**) as well as in HCC4006 stable cell lines that were treated with Z-guggulsterone (40 μM) (**E**). Quantitative RT-PCR was performed to evaluate the FXR, SHP, and cyclin D1 mRNA abundance in H1975 cells that were treated with Z-guggulsterone (40 μM) (**F**) or transfected with NC- or FXR-siRNA sequences (**G**) as well as in HCC4006 stable cell lines that were treated with Z-guggulsterone (40 μM) (**H**). The cell proliferation rate of H1975 (**I**) and H1299 (**J**) cells was determined by the SRB assay three days after transfection with NC- or SHP-siRNA sequences. Western blot analysis was performed to evaluate the expression of SHP, FXR, cyclin D1, and p-Rb in H1975 (**K**) and H1299 (**L**) cells that were transfected with NC- or SHP-siRNA sequences. The data are presented as the mean ± SD of at least three independent experiments. *p < 0.05, compared with the control, NC, or mock group, respectively; ^†^ *p* < 0.05, compared with the FXR-overexpressed HCC4006 cell group.

Cell cycle arrest and apoptosis are considered two primary approaches in proliferation inhibition. We examined the effects of FXR on cell cycle and apoptosis in NSCLC cells. Both Z-guggulsterone and FXRsiRNAs increased the proportion of H1975 cells in the G0/G1 phase, and decreased the proportion in the S and G2/M phases (Fig. 4A and B). Similar trends were found in H1299 cells that underwent the same treatment (Supplementary Fig. S1A and B), indicating a critical role for FXR in the G1/S transition. However, no apparent change in apoptosis was observed in H1975 and H1299 cells upon either Z-guggulsterone or FXRsiRNAs treatment (Supplementary Fig. S1G and H).

Certain regulators acting in the cell cycle G1/S transition were subsequently evaluated by western blot analysis^18^. As shown, the cyclin D1, CDK2, CDK4, CDK6, and phosphorylated Rb (p-Rb) protein levels were remarkably reduced in H1975 cell that were treated with Z-guggulsterone, while only cyclin D1 and p-Rb proteins were down-regulated by FXRsiRNAs (Fig. 4C and D). Similar changes were also found in H1299 cells (Supplementary Fig. S1C and D). In contrast, ectopic FXR expression in HCC4006 increased the cyclin D1 and p-Rb protein levels (Fig. 4E). As a result, the influences of Z-guggulsterone on CDK2, CDK4, and CDK6 expression could be considered nonspecific^19^. Quantitative RT-PCR analysis demonstrated consistent results that both Z-guggulsterone and FXRsiRNAs reduced the cyclin D1 mRNA level in H1975 (Fig. 4F and G) and H1299 cells (Supplementary Fig. S1E and F), whereas overexpression of FXR increased the cyclin D1 abundance in HCC4006 (Fig. 4H). Since cyclin D1 is required for Rb phosphorylation by CDK4/6 in cell cycle initiation^20^, we speculated that FXR might function as a positive regulator of cyclin D1 transcription and G1/S transition.

Furthermore, we addressed whether the effects of FXR in NSCLC depend on its downstream effector, SHP^9^. There was no detectable change in either *in vitro* proliferation or cyclin D1 and p-Rb expression in H1975 and H1299 cells that were transfected with SHPsiRNAs (Fig. 4I–L). These findings imply that FXR induces cell proliferation and cyclin D1 expression through a mechanism that is independent of SHP in NSCLC.

### FXR is recruited to the cyclin D1 promoter and promotes its transcription

**Figure 5:**
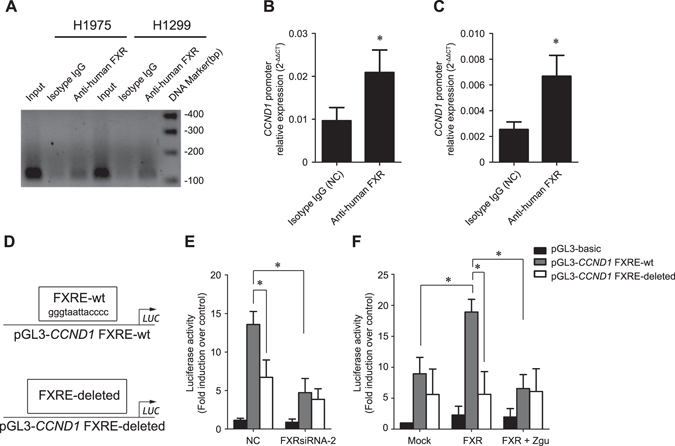
FXR is recruited to the cyclin D1 promoter and promotes its transcription. A ChIP assay was performed in H1975 and H1299 cells with anti-human FXR mouse monoclonal antibody and primer sets described in the “Materials and methods” section. The isotype IgG was used as a negative control (NC), and the input chromatin sample was used as a positive control. (**A**) Representative PCR amplification products are shown. Enrichment of FXR protein in the putative motif of *CCND1* promoter in H1975 (**B**) and H1299 (**C**) cells was determined relative to input samples, respectively. (**D**) Schematic representation of a wild-type *CCND1* reporter plasmid (pGL3-*CCND1* FXRE-wt) and an FXRE-deleted *CCND1* reporter plasmid (pGL3-*CCND1* FXRE-deleted). (**E**) H1975 cells were transfected with NCsiRNA or FXRsiRNA-2; (**F**) HCC4006 stable cell lines were treated with or without Z-guggulsterone (Z-gu, 40 μM). One day later, all cells were transfected with the indicated reporter plasmids and then collected for measurements of the luciferase activities at 24 h post-transfection. The data were presented as the mean ± SD of at least three independent experiments. *p < 0.05.

FXR is a transcription factor that controls target gene transcription through binding to an FXR-responsive element (FXRE)^21^. Gene sequence analysis revealed that the −2000/+209 region of the human *CCND1* promoter contains a putative inverted repeat-0 FXRE sequence (GGGTAATTACCCC) at nucleotide −1539 from the transcription initiation site. To confirm the hypothesis that FXR might transactivate *CCND1*, a chromatin immunoprecipitation (ChIP) assay was performed. Its results showed that clear bands could be amplified from DNA samples immunoprecipitated by anti-FXR antibody in H1975 and H1299 cells, while no band was amplified in the negative controls (NC, isotype IgG) (Fig. 5A–C), indicating that FXR can directly bind to the putative motif in the *CCND1* promoter.

We performed a luciferase reporter assay using luciferase reporter plasmids harboring the *CCND1* promoter region with or without a wild-type FXRE sequence (Fig. 5D). To achieve more effective knockdown of FXR expression, only FXRsiRNA-2 was used. We observed that wild-type *CCND1* promoter activity was significantly higher than that of the FXRE-deleted *CCND1* promoter in H1975 cells (Fig. 5E). FXR knockdown in H1975 significantly reduced wild-type *CCND1* promoter activity without reducing FXRE-deleted *CCND1* promoter activity. Conversely, ectopic overexression of FXR in HCC4006 increased the wild-type *CCND1* promoter activity by approximately 100% compared to the mock group, whereas deletion of the FXRE motif repressed this elevation (Fig. 5F). Z-guggulsterone retained the activity of the wild-type *CCND1* promoter, rather than the FXRE-deleted ones, in FXR-overexpressed HCC4006 cells. These findings corroborated the contribution of FXR in directly activating cyclin D1 transcription in NSCLC.

### Overexpression of cyclin D1 rescues NSCLC cells from the antiproliferative effects induced by FXR suppression

**Figure 6:**
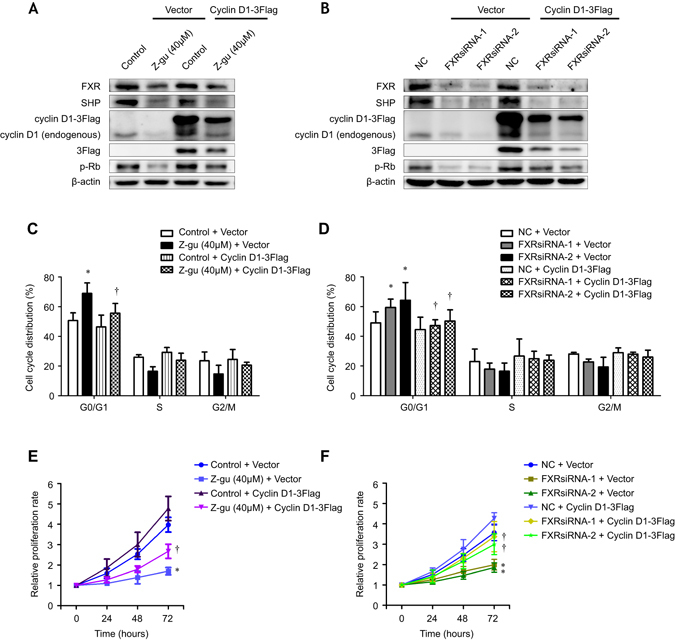
Overexpression of cyclin D1 rescues cell proliferation defects in FXR-suppressed NSCLC cells. Western blot analysis was performed to evaluate the expression of FXR, SHP, endogenous and ectopic cyclin D1, 3Flag, and p-Rb in H1975 cells that were treated with Z-guggulsterone (Z-gu, 40 μM) (**A**) or transfected with NC- or FXR-siRNA sequences (**B**), in addition to the transfection with vector or cyclin D1-3Flag plasmid. 3Flag expression represented ectopic cyclin D1 expression. The cell cycle profile of H1975 cells was analyzed by flow cytometry two days after the treatment with Z-guggulsterone (40 μM) with or without transfection of cyclin D1-3Flag (**C**), or the cotransfection with FXRsiRNAs and/or cyclin D1-3Flag plasmid (**D**). The cell proliferation rate of H1975 cells was determined after the treatment with Z-guggulsterone (40 μM) with or without transfection of cyclin D1-3Flag (**E**), or the cotransfection with FXRsiRNAs and/or cyclin D1-3Flag plasmid (**F**) by using SRB assay at different time points. All experiments were repeated at least three times. The data were shown as the mean ± SD. *p < 0.05, compared with the control + vector or NC + vector group, respectively; ^†^ *p* < 0.05, compared with the Z-guggulsterone (40 μM) + vector or FXRsiRNAs + vector group.

To examine the functional relevance of FXR and its target cyclin D1, rescue experiments were conducted. 3Flag-tagged cyclin D1 was forcefully expressed into H1975 and H1299 cells treated with either Z-guggulsterone or FXRsiRNAs, respectively; the effects on cell cycle distribution and *in vitro* proliferation were determined. Western blot analysis showed that cyclin D1 and p-Rb, both substantially reduced in Z-guggulsterone or FXRsiRNAs treated H1975 (Fig. 6A and B) and H1299 cells (Supplementary Fig. S2A and B), respectively, were increased in cells ectopically expressing cyclin D1. As depicted, overexpression of cyclin D1 partially reversed the delayed G1/S transition and the impaired *in vitro* proliferation induced by Z-guggulsterone in H1975 cells (Fig. 6C and E). Similar changes were also found in H1299 upon the same treatment (Supplementary Fig. S2C and E). Moreover, FXRsiRNAs-induced G0/G1 arrest and cell proliferation inhibition in H1975 and H1299 cells were almost completely abrogated in cells transfected with cyclin D1-3Flag, compared to vector control (Fig. 6D,F, and Supplementary Fig. S2D, F). These results clearly demonstrated that upregulation of cyclin D1 expression was responsible for the proliferation-promoting function of FXR in NSCLC.

### Correlation analysis of FXR and cyclin D1 expression in NSCLC specimens

**Figure 7:**
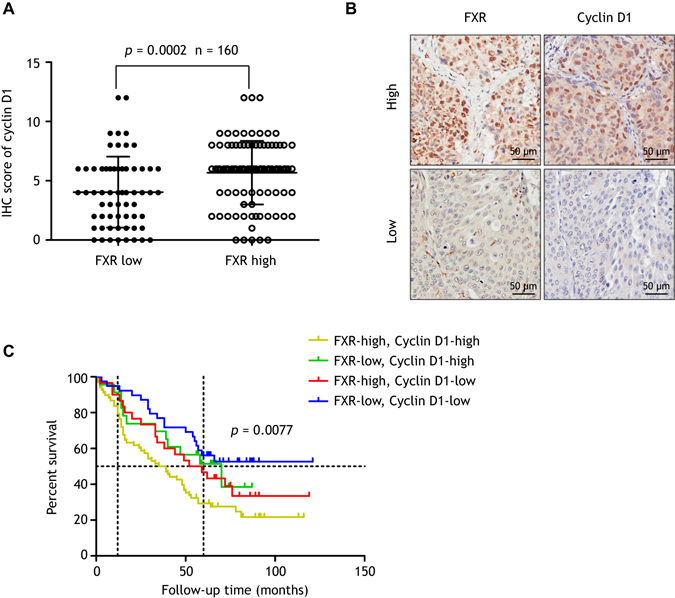
FXR and cyclin D1 expression are positively correlated in NSCLC samples. Cyclin D1 expression was assessed by IHC in the same cohort of specimens. (**A**) The IHC score of cyclin D1 is increased in “FXR high” tumors compared to “FXR low” tumors (*p* = 0.0002). (**B**) Representative images of high (upper panel) or low (lower panel) expression of FXR and cyclin D1 in serial sections of NSCLC specimens. (**C**) Kaplan-Meier analysis showing that patients with both FXR and cyclin D1 high expression have the worst OS (*p* = 0.0077).

To further confirm the association between FXR and cyclin D1 in NSCLC, we assessed cyclin D1 expression by IHC in the same cohort of specimens. We detected significantly higher cyclin D1 levels in NSCLC compared to corresponding pericarcinous lung tissues (Supplementary Fig. S3A). Kaplan-Meier analysis revealed a significantly shorter OS in “cyclin D1 high” (scores 6–12) NSCLC patients than in “cyclin D1 low” (0–4) patients (*p* = 0.0113) (Supplementary Fig. S3B), which was consistent with previous reports^22, 23^. Additionally, cyclin D1 protein was increased in “FXR high” tumors (Fig. 7A and B), and chi-square analysis demonstrated a positive correlation between FXR and cyclin D1 expression in NSCLC specimens (*p* < 0.001) (Table 4). Notably, a combinatorial expression pattern of “FXR high” and “cyclin D1 high” predicted the worst OS in NSCLC patients (*p* = 0.0077) (Fig. 7C).

**Table 4:** Correlation analysis between FXR and cyclin D1 protein expression in NSCLC.

NSCLC samples	Cyclin D1		Correlation coefficient	*p*-value
	Low	High		
FXR low	39	23	0.318	<0.001^a^
FXR high	30	68		

^a^Data were analyzed using chi-square test.

## Discussion

In this study, we identified FXR as a novel proto-oncogene that is markedly upregulated in NSCLC. Our data indicated that FXR contributes to tumor growth via directly transactivating *CCND1* and then promoting cell cycle progression. Furthermore, the expression of FXR and cyclin D1 was found to be positively correlated in NSCLC samples and could cooperatively contribute to patient sub-stratification. To the best of our knowledge, this study is the first to comprehensively analyze the expression of FXR in NSCLC, as well as assess the functional role in carcinogenesis.

FXR, an essential regulatory nuclear receptor for maintaining BA homeostasis, is deregulated in many malignancies^8^. It is well established that FXR deficiency leads to spontaneous liver tumors and increased colon cell proliferation in mice as well as increased small intestine adenocarcinoma formation in APC^min^ mice, supporting a tumor-suppressive role of FXR in these cancer types^10, 12^. However, Guan *et al*. reported that FXR is overexpressed in esophageal adenocarcinomas, and that knockdown of FXR causes tumor cell growth inhibition both *in vitro* and *in vivo* ^14^. Research on breast cancer has demonstrated that activation of FXR promotes cell proliferation within ER-positive cancer cells, indicating that this receptor likely has an oncogenic function^15^. In the present study, we provide compelling clinical evidence that in NSCLC, FXR is significantly increased and independently predicts poor outcomes. Additionally, FXR promoted NSCLC cell proliferation and tumorigenicity in both cultured cells and animal models, indicating that FXR could act as an oncogene in NSCLC development. These findings further highlight a dual role of FXR as either a tumor-suppressor or an oncogenic protein, suggesting the need to dissect its precise roles in different cancer types. The genetic and environmental cues are believed to be critical to determine the function of FXR in a defined context.

Specific ways by which FXR exerts proliferation-promoting functions in NSCLC were subsequently explored. Cell cycle arrest and apoptosis are considered two primary approaches underlying proliferation inhibition in cancer cells. We found that either pharmacological inhibition or knockdown of FXR delayed the NSCLC cell cycle G1/S transition. Interestingly, no detectable change in apoptosis was noted. Since the FXR antagonist guggulsterone was reported to induce cell apoptosis in esophageal cancer, pancreatic cancer, and head and neck cancer, among others, such discordance might be ascribed to the specific intrinsic characteristics across different tumor types^14, 24, 25^. Further experiments have been performed to assess the involvement of cell cycle regulators in the FXR-driven G1/S transition^18^. Cyclin D1, encoded by *CCND1*, is a well-documented regulator of the G1/S transition through activating CDK4/6 kinase and subsequently phosphorylating Rb^20^, that was dramatically reduced at both the protein and mRNA levels in the FXR-knockdown NSCLC cells, while it was increased in ectopic FXR-overexpressing cells. Our results support a reasonable mechanism that FXR facilitates NSCLC cell cycle G1/S transition via upregulation of cyclin D1 transcription. In accordance with our findings, cyclin D1 expression was concomitantly lower in hepatocyte-specific FXR knockout mice than in wild-type mice after partial hepatectomy in a previously published study^26^. However, we found that knockdown of SHP, a well-known FXR target gene mediating several major functions of FXR^8, 9^, neither affected NSCLC cell proliferation nor caused changes in cyclin D1 or p-Rb expression, indicating, at least in this context, that FXR-induced cell proliferation is SHP independent.

As a transcription factor, FXR regulates target gene expression by binding, either as a monomer or as a heterodimer with RXRα, to an FXRE sequence^27^. Many FXR target genes have been characterized, and they are involved in a variety of pathophysiological processes, including tumorigenesis^28–31^. In this study, we demonstrated for the first time that FXR is recruited to the *CCND1* promoter in NSCLC cells and activates its transcription. Furthermore, overexpression of cyclin D1 in FXR-suppressed NSCLC cells restored the cell cycle progression and *in vitro* proliferation, suggesting that FXR promotes NSCLC cell growth via the upregulation of cyclin D1 expression. Notably, we observed a positive correlation between the expression of FXR and cyclin D1 in NSCLC samples, and the shortest OS was observed in patients with both high FXR and high cyclin D1 expression, indicating a potential mechanistic link. In agreement with our findings, *CCND1* is a well-recognized oncogene, as well as a poor prognostic indicator, that is overexpressed in many cancers, including NSCLC^22, 23^. Here, we provide a new biomarker, FXR, which, in combination with cyclin D1, more effectively sub-stratifies NSCLC patients. Moreover, recent research has shown that the therapeutic outcomes of targeted drugs are largely determined by the response of downstream effectors to the corresponding treatments^32–34^. This study supports the development of FXR-targeted therapies for NSCLC and enables the downstream effector cyclin D1 to act as a candidate probe for response or resistance to FXR-based treatments.

Altogether, we found that FXR acts as a novel proto-oncogene in NSCLC via the target gene *CCND1*, driving cell cycle progression and tumor growth. The current study extends our understanding of the function for FXR in NSCLC tumorigenesis, providing a promising prognostic biomarker and therapeutic approach.

## Materials and Methods

### Patients and tissue microarray

Primary NSCLC and corresponding pericarcinous lung tissues were consecutively collected from the Department of Pathology in Ren Ji Hospital (Shanghai, China) between 2008 and 2010. Tissue microarrays (TMAs) were constructed as previously described^35^. For each sample, two cores with a 1.6-mm diameter were obtained from the original paraffin block. Clinicopathological information was retrospectively reviewed. The histology was determined according to the criteria of the World Health Organization, and pathological stages were classified according to the seventh edition of the lung cancer staging system^36^. Survival data were recorded on the basis of follow-up clinic visits or telephone calls. Patients who had incomplete clinical or follow-up data or who had received neoadjuvant chemotherapy or radiotherapy were excluded. In total, 160 patients were included. This study was approved by the Ethics Committee of Ren Ji Hospital, and informed consent was obtained from all participants. All methods were performed in accordance with the approved guidelines of School of Medical graduate Shanghai Jiao Tong University.

### Immunohistochemical (IHC) analysis

IHC staining for FXR and cyclin D1 expression in TMAs was performed as described^4^. Anti-bile acid receptor (*NR1H4*) antibody (Abcam, Cambridge, MA) and anti-cyclin D1 antibody (Cell Signaling Technology, Beverly, MA) were applied at 1:100 and 1:50, respectively. Concentration-matched nonspecific rabbit IgG was used as a isotype control. Two trained pathologists viewed the IHC staining results and reached a final consensus. The scoring for the staining intensity was as follows: negative (0), weak (1), moderate (2) and intense (3). The scoring for the percentage of positive cells was as follows: 0% (0), 1–25% (1), 26–50% (2), 51–75% (3) and 76–100% (4). The final FXR or cyclin D1 IHC score was obtained by multiplying the intensity and percentage scores, which were defined as low (including scores of 0–4) or high (6–12) expression.

### Cell culture and cell proliferation assays

Human NSCLC cell lines (H1650, H460, HCC4006, H1975 and H1299), and a hepatoma cell line (HepG2) were purchased from the American Type Culture Collection (ATCC). All cells were cultured in RPMI-1640 or DMEM-F12 (Invitrogen Corporation, Carlsbad, CA) supplemented with 10% FBS. The *in vitro* cell proliferation was assessed by sulforhodamine B (SRB) assay as previously described^37^. The absorbance was measured at 560 nm in a microplate reader.

### Western blot

Western blot analysis was performed according to previously described procedures^16^. We used primary antibodies, including anti-bile acid receoptor (*NR1H4*) antibody, anti-SHP antibody (Santa Cruz, CA), anti-phospho-Rb antibody, anti-cyclin D1 antibody, anti-cyclin E1 antibody, anti-CDK2 antibody, anti-CDK4 antibody, anti-CDK6 antibody, anti-p21^Waf1/Cip1^ antibody, anti-p27^Kip1^ antibody, and anti-β-actin antibody (Cell Signaling Technology) according to the manufacturer’s recommended dilutions.

### Quantitative real-time PCR (RT-PCR) analysis

Quantitative RT-PCR was performed as previously described^16^. Primers were designed as follows: FXR forward, 5′-GATTGCTTTGCTGAAAGGGTC-3′; reverse, 5′-CAGAATGCCCAGACGGAAG-3′; SHP forward, 5′-AGGCCTCCAAGCCGCCTCCCACATTGGGC-3′; reverse, 5′-GCAGGCTGGTCGGAAACTTGAGGGT-3′; cyclin D1 forward, 5′-CGTGGGCTCTAAGATGAAGG-3′; reverse, 5′-TGCGGATGATCTGTTTGTTC-3′; β-actin forward, 5′-TTGCTGATCCACATCTGCT-3′; reverse, 5′-GACAGGATGCAGAAGGAGAT-3′.

### Transfection

Cells were transfected with siRNA by using Lipofectamine RNAiMAX reagent (Invitrogen Corporation) according to the manufacturer’s instructions. The target sequences of double-stranded nucleotides used in siRNA transfection were 5′-GGACCATGAAGACCAGATT-3′(FXRsiRNA-1) and 5′-GTAGCAGAGATGCCTGTAA-3′(FXRsiRNA-2) for FXR knockdown, 5′-GGAATATGCCTGCCTGAAA-3′(SHPsiRNA-1) and 5′-TCGCCCTATCATTGGAGAT-3′(SHPsiRNA-2) for SHP knockdown, and 5′-TTCTCCGAACGTGTCACGT-3′ as a negative control (NC) (RiboBio, Guangzhou, China). To perform rescue experiments, cells were transfected with pcDNA3.1(+)-cyclin D1-3Flag plasmids, constructed with OBiO Biotechnology (Shanghai, China), by using Lipofectamine 2000 (Invitrogen Corporation) according to the manufacturer’s protocol. Empty pcDNA3.1(+)-3Flag plasmid was used as control.

### Construction of lentiviral vectors and cell infection

Lentiviral vectors were constructed with OBiO Biotechnology (Shanghai, China), using either pCMV-NR1H4-PGK-PuroR plasmids, which carry the full-length *NR1H4* coding sequence (GenBank accession NM_005123.3), or pCMV-G&PR-U6-shRNA plasmids, which generate shRNA targeting FXR or NCshRNA as described above. The detailed methodology for cell infection is provided in the Supplementary Information.

### Confocal immuofluorescence

After treatment, cells were grown on cover-slips with 1% gelatin for 24 h. Confocal immuofluorescence was performed as previously described^16^. Anti-human Ki-67 antibody (Spring Bioscience, Fremont, CA) was applied at 1:100.

### Tumorigenicity assays in nude mice

The animal experiments were performed in accordance with the guidelines of the Experimental Animal Ethics Committee of Shanghai Jiao Tong University. All experimental procedures were approved by the Experimental Animal Ethics Committee of Shanghai Jiao Tong University. The detailed methodology is provided in the Supplementary Information.

### Cell cycle and apoptosis analysis by flowcytometry

For cell cycle analysis, cells were collected 48 h after treatment. The cell cycle distribution was measured using a flow cytometer (BD Bioscience, San Jose, CA). For apoptosis analysis, cells were harvested with EDTA-free trypsin 72 h after treatment and stained using a FITC Annexin V Apoptosis Detection Kit (BD Bioscience) according to the manufacturer’s instructions. The stained cells were examined with a flow cytometer.

### Luciferase reporter assay

Luciferase reporter plasmids carrying either wild-type or FXRE-deleted *CCND1* promoter sequence were generated by OBiO Biotechnology (Shanghai, China). The detailed methodology is provided in the Supplementary Information.

### Chromatin immunoprecipitation (ChIP) assay

The ChIP assay was conducted using a SimpleChIP Plus Enzymatic Chromatin IP Kit (Cell Signaling Technology) according to the manufacturer’s instructions. The detailed methodology is provided in the Supplementary Information. Primers used in quantitative RT-PCR were as follows: cyclin D1 promoter, forward 5′-TAGGTGCTCCCTGCTGGGGC-3′; reverse 5′-ACCTTTCAGGGTGAATTCCTCCC-3′.

### Statistical analysis

Comparisons between groups were performed using Student’s *t* test or the Mann-Whitney *U* test. Correlational analyses were conducted using the chi-square test or Fisher’s exact test. Survival data were analyzed by the Kaplan-Meier method (log-rank test). A multivariate Cox regression model was used to identify independent predictors. Statistical analyses were performed using SPSS 17.0 (SPSS Inc., USA). A value of *p* < 0.05 was considered statistically significant.

## Electronic supplementary material

Supplementary information
