# Supplementary material for: Farnesoid X receptor, a novel proto-oncogene in non-small cell lung cancer, promotes tumor growth via directly transactivating CCND1
Source: Sci Rep. 2017 Apr 4;7:591. doi: 10.1038/s41598-017-00698-4 (PMC5428828; doi:10.1038/s41598-017-00698-4)

Farnesoid X receptor, a novel proto-oncogene in non-small cell lung cancer, promotes tumor growth via directly transactivating *CCND1*

Wenjie You[1,a], Bi Chen[2,a], Xueqing Liu[1,a], Shan Xue[1], Hui Qin[1] & Handong Jiang[1,*]

Supplementary information

Materials and methods

Cell infection

For stable FXR overexpression, HCC4006 cells were cultured in 6-well plates (2.5 × 10^5 cells) with antibiotic-free medium for 24 h; then, they were infected with lentiviral pCMV-NR1H4-PGK-PuroR or the corresponding control lentivirus at the multiplicity of infection (MOI) of 20 pfu/cell. For stable knockdown of FXR, H1975 cells were also seeded into 6-well plates (2 × 10^5 cells) and were then infected with lentiviral vectors expressing FXR- or NC-shRNA at the MOI of 20 pfu/cell. The selection of stably-transfected cells was performed 48 h later with 1 μg/mL puromycin (Invitrogen), and the infection efficiency was determined by western blot and quantitative RT-PCR.

Tumorigenicity assays in nude mice

Briefly, eighteen 6-week-old male BALB/c nude mice were randomly divided into three groups (H1975 with NCshRNA, FXRshRNA-1 and FXRshRNA-2), and subcutaneously injected in the right flank with the corresponding logarithmic phase cells (5 × 10^6 cells/100 μL per mouse). The tumor size and body weight were measured twice per week. Tumor volumes were calculated as follows: volume = maximum diameter × minimum diameter^2 × 0.5. Thirty-two days later, all mice were sacrificed, and tumors were photographed and weighed after separation.

Luciferase reporter assay

Briefly, H1975 cells transfected with NCsiRNA or FXRsiRNA-2 and HCC4006 stable cell lines treated with or without Z-guggulsterone (40 μM) were cultured in 24-well

plates (4×10^4 cells) with antibiotic-free medium for 24 h. Then, they were transfected with luciferase reporter plasmids harboring wild-type or FXRE-deleted *CCND1* promoter sequences or basic vectors using Lipofectamine 2000 reagent (Invitrogen Corporation). The renilla luciferase reference plasmid was included in each transfection system as an internal control to normalize the transfection efficiency. The luciferase activities were measured at 24 h post-transfection with the Dual-Luciferase Reporter Assay System (Promega, Madison, WI) using a luminometer (Promega) according to the manufacturer's protocol. Three independent experiments were conducted in duplicate.

ChIP assay

In brief, H1975 and H1299 cells (1×10^7) were cross-linked with 1% formaldehyde, quenched with 0.125M glycine and lysed with SDS-containing buffer. The gathered chromatin DNA was treated with micrococcal nuclease and then sonicated to an average size of 100-1000 bp. The fragmented chromatin was aliquoted as input DNA, or was immunoprecipitated with IgG or anti-human FXR mouse monoclonal antibody (Perseus Proteomics Inc., Tokyo, Japan) at 4°C overnight. The immunocomplexes were pulled down with protein G magnetic beads, de-crosslinked, and purified as described. The eluted samples and corresponding input DNA were analyzed by quantitative RT-PCR. Finally, the PCR products were separated by electrophoresis on a 1% agarose gel and visualized in the Image Lab Analysis System (Bio-Rad, Hercules, CA).

Figure legends

Figure S1.

Effects of FXR on the cell cycle distribution, cell cycle regulators and apoptosis in NSCLC. H1975 and H1299 cells were treated with Z-guggulsterone (Z-gu, 40 μM) or transfected with NC- or FXR-siRNA sequences. (A and B) cell cycle profile in H1299 cells is summarized. Western blot (C and D) and quantitative RT-PCR (E and F) were performed to evaluate the expression levels of FXR, SHP and the indicated cell cycle regulators in H1299 cells after treatment. Cell apoptosis was analyzed by flow

cytometry after staining with Annexin V and PI. No change in the percentages of apoptotic cells was detected in H1975 (left graph) and H1299 (right graph) cells that were treated with Z-guggulsterone (40 μM) (G) or transfected with NC- or FXR-siRNA sequences (H). All experiments were repeated at least three times. * $p < 0.05$, compared with the control or NC group, respectively.

Figure S2.

Forced cyclin D1 expression rescues cell proliferation defects in FXR-suppressed H1299 cells. H1299 cells were treated with Z-guggulsterone (Z-gu, 40 μM) or transfected with NC- or FXR-siRNA sequences, in addition to the transfection with vector or cyclin D1-3Flag plasmid. (A, B) Western blot analysis was performed to evaluate the expression of FXR, SHP, endogenous and ectopic cyclin D1, 3Flag, and p-Rb in H1299 cells after the indicated treatment. (C, D) The cell cycle profile of H1299 cells was analyzed by flow cytometry two days after the indicated treatment. (E, F) The cell proliferation rate of H1299 cells was determined after the indicated treatment by using SRB assay at different time points. The data were presented as the mean ± SD of at least three independent experiments. * $p < 0.05$, compared with the control + vector or NC + vector group, respectively; † $p < 0.05$, compared with the Z-guggulsterone (40 μM) + vector or FXRsiRNAs + vector group.

Figure S3.

Cyclin D1 expression is increased in NSCLC specimens and predicts poor patient survival. (A) IHC score of cyclin D1 in 160 pairs of NSCLC and pericarcinous lung tissues ($p < 0.001$). (B) “Cyclin D1 high” patients (n = 91) have a shorter OS than “cyclin D1 low” patients (n = 69) ($p = 0.0113$).

Supplementary Figure S1

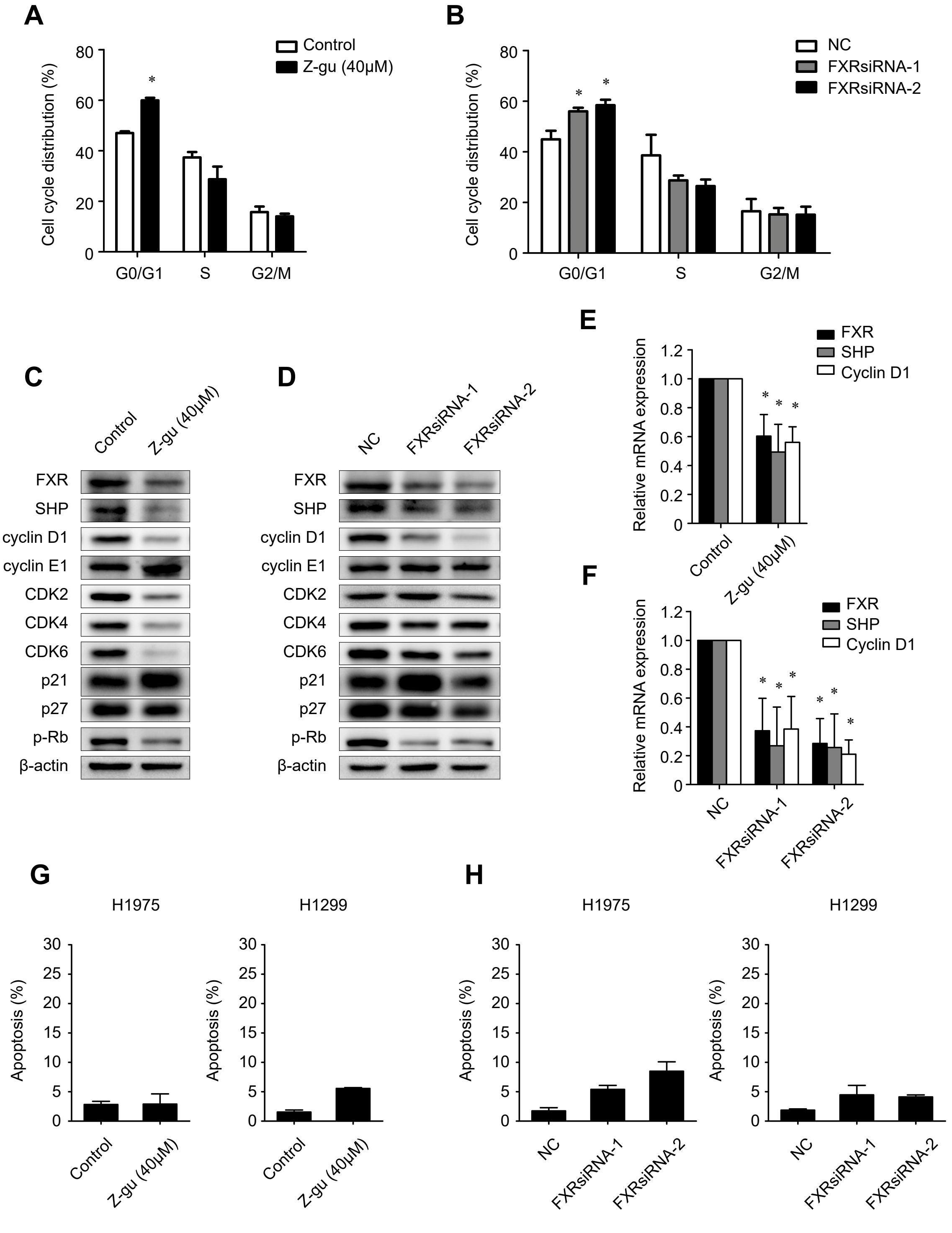

Supplementary Figure S2

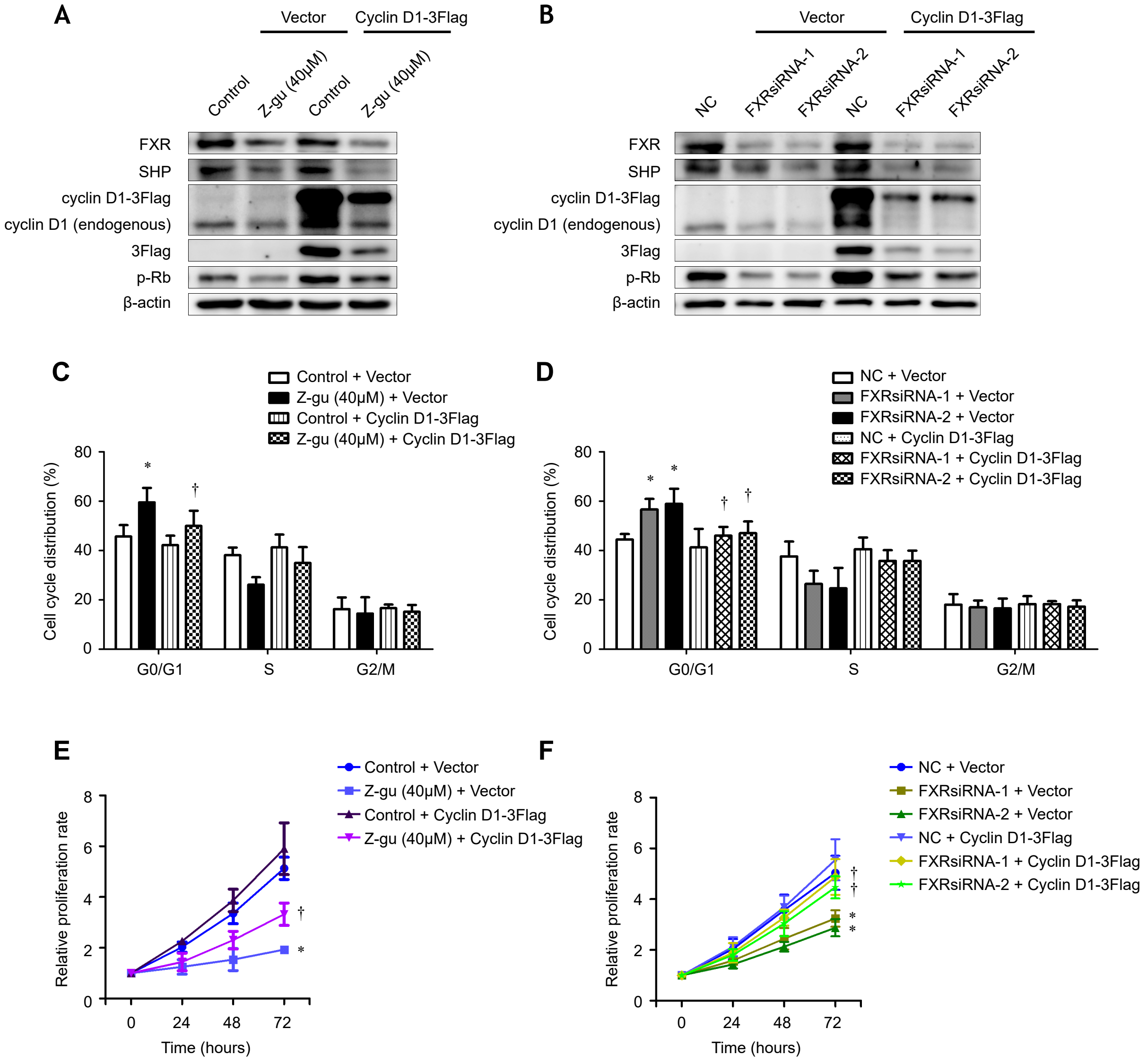

Supplementary Figure S3

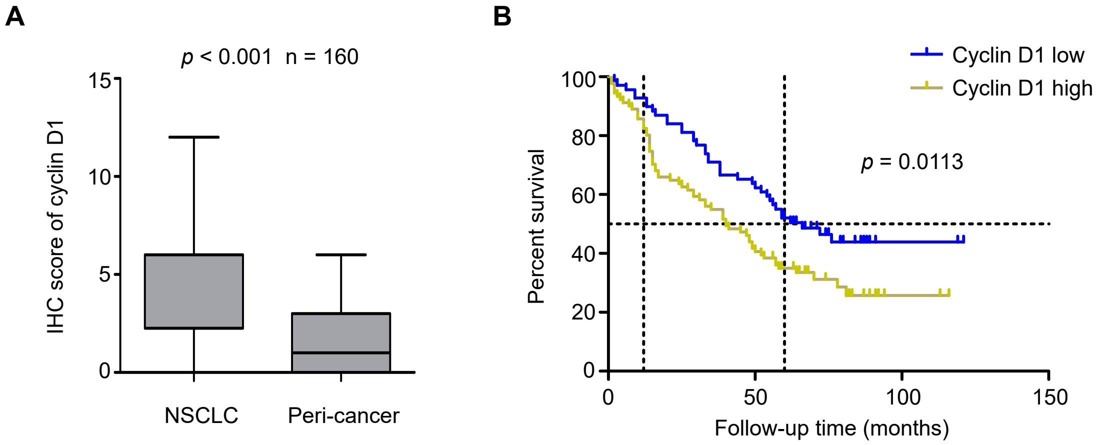

Supplement: Supplementary file 1 — Supplementary information [file 41598_2017_698_MOESM1_ESM.pdf]
